# Occupational injuries among healthcare workers: a nationwide study in Turkey

**DOI:** 10.3389/fpubh.2024.1505331

**Published:** 2024-12-05

**Authors:** İrem Medeni, Mehmet Erdem Alagüney, Volkan Medeni

**Affiliations:** ^1^Employee Health Department, General Directorate of Public Health, Ministry of Health, Ankara, Türkiye; ^2^Department of Public Health, Faculty of Medicine, Gazi University, Ankara, Türkiye

**Keywords:** occupational injury, healthcare workers, occupational safety, sharps injuries, community-based participatory research

## Abstract

**Introduction:**

The health sector is a field where employees are frequently exposed to occupational injuries due to high-risk working conditions. This study aimed to examine the distribution and causes of occupational injuries experienced by healthcare workers in the last 5 years in Turkey.

**Materials and methods:**

In this population-based and national-scale study, occupational injuries reported to the Ministry of Health from healthcare organizations in 81 provinces of Turkey between 01.01.2019 and 31.12.2023 were retrospectively analyzed. Variables such as age, gender, title, place of employment, types of injuries, causes, and outcomes of occupational injuries were evaluated.

**Results:**

A total of 68,563 occupational injuries were reported between the years analyzed. 64.5% of the injuries affected female workers. Occupational injuries occurred most frequently during the summer months and in hospitals. According to age groups, the highest rate of occupational injuries was observed in the 20–29 age group with 39.3%. Among the types of occupational injuries, sharps injuries were the most common, with 55.3%. It was followed by slips, trips, and falls (13.2%). As a result of occupational injuries, 76.2% of healthcare workers were able to return to work without long-term absence. Over the 5 years, 61 healthcare workers lost their lives due to occupational injuries. Nurses and midwives were the occupational groups most exposed to injuries, followed by cleaning staff.

**Conclusion:**

Turkey's healthcare workers have a high exposure rate to occupational injuries. Women and young workers are the most affected groups. Strengthening the occupational safety culture and providing safe working environments is necessary.

## 1 Introduction

According to the International Labor Organization, an occupational injury is “an unexpected and unplanned event, including acts of violence, arising out of or in connection with work, which causes injury, illness or death to one or more workers” (1). These injuries can range from minor incidents, such as cuts and bruises, to significant life-threatening situations. They can cause human suffering, lost productivity, and substantial financial losses (2). Every 15 s, somewhere in the world, one worker dies from an occupational injury or work-related illness, and 153 workers are injured on the job (3). The indirect costs of work injuries or occupational diseases, including lost working time, compensation, production interruptions, and medical expenses, are 4–10 times higher than their direct costs and amount to about 4% of global gross national product, about $2.8 trillion (4).

The health sector comprises many employees working in non-standard work schedules (5). Healthcare workers work in hazardous environments with varying degrees of exposure to physical, biological, chemical, and psychological factors (6). Healthcare workers face a higher risk of injury than workers in other sectors. Cuts, strains, sprains, fractures, and common traumas, which usually occur with risks such as sharps injuries, slips, trips, falls, violence, overexertion, and patient handling activities, emphasize the need to increase awareness and preventive measures to reduce workplace hazards (7, 8). Occupational injuries stem from environmental, procedural, and managerial factors that may contribute to unsafe conditions and behaviors. These injuries can impact individuals, families, communities, and societies by affecting healthcare workers' physical and mental wellbeing, as well as the quality of healthcare services provided (9, 10).

Since healthcare workers have a crucial role in protecting public health, it is essential to protect them from occupational injuries and to ensure they work in safe workplaces (11). Therefore, examining occupational injuries of healthcare workers is valuable to understanding this occupational group's unique needs and risks and may help to take appropriate protective measures. Identifying the prevalence, distribution characteristics, and primary causes of occupational injuries can support both individual and organizational interventions, such as safer working environments, improved training, appropriate staffing, and enhanced supervision. These measures may reduce occupational injuries, increase productivity, and help provide better quality services while minimizing associated cost (12). In this context, examining occupational injuries in healthcare workers plays a critical role in the sustainability and efficiency of healthcare services.

In Turkey, occupational injuries are legally recorded by the Social Security Institution, which operates under the Ministry of Labor and Social Security. This institution publishes annual reports on occupational injury data. However, the data collection related to occupational injuries involving healthcare workers began in August 2018, under the Ministry of Health's Department of Employee Health. Our study aims to analyze these data and provide a detailed examination of the distribution of occupational injuries affecting healthcare workers in Turkey over the past 5 years. This nationwide study will make a significant contribution to efforts aimed at improving occupational health and safety.

## 2 Materials and methods

This study, based on national data, analyzes occupational injuries among healthcare workers reported to the Occupational Diseases and Injuries Surveillance Unit of the Ministry of Health's General Directorate of Public Health from January 1, 2019, to December 31, 2023. Health service providers affiliated with the Ministry of Health in 81 provinces of Turkey report the occupational injuries of healthcare workers to the relevant unit monthly through the Occupational Injury Monthly Assessment Form. These institutions are state hospitals, training and research hospitals, university hospitals, emergency health services, family health centers, oral and dental health centers, provincial health directorates, district health directorates, and community health centers. Occupational injury files sent to the ministry via electronic mail were included in the evaluation.

This retrospective, nationwide, and observational study included all files submitted to the relevant institution within the specified date range. The variables in the study were age, gender, title, place of employment, injury date, injury type, possible causes of the injury, consequences of occupational injuries, and notifying health institutions.

All occupational injuries were categorized according to years and months. Healthcare workers with occupational injuries were grouped according to age as < 20, 20–29, 30–39, 40–49, 50–59, and 60 and over.

The consequences of occupational injuries experienced by healthcare workers are return to work without treatment or after outpatient treatment, medical leave due to temporary incapacity, inpatient treatment, having to leave work, and death.

The types of occupational injuries involving healthcare workers include sharps injuries, slips, trips and falls, bumps and collisions, biological exposure, violence, traffic accidents, chemical exposure, burns, electric shocks, explosions, and other injuries. Possible causes of occupational injuries are divided into unsafe behaviors and conditions. Unsafe behaviors include physical and mental fatigue, failure to use equipment, inappropriate working speed, failure to follow instructions, failure to use personal protective equipment, unauthorized work, undisciplined work, and improper lifting. Unsafe conditions are improper stacking, fire hazard, explosion hazard, inappropriate or defective equipment, inappropriate or missing personal protective equipment, inappropriate weather conditions, lack of warnings and alerts, inadequate lighting, and noise.

In this study, we have addressed unsafe behaviors and conditions as contributing factors rather than direct causes of injuries. Emphasizing these factors does not imply that they are the sole cause of occupational injuries. On the contrary, our goal is to highlight the importance of individual responsibility and awareness within a broader occupational safety culture. We believe that improving occupational safety requires a comprehensive approach that addresses both individual characteristics and institutional and governmental policies.

The job titles of healthcare workers who had occupational injuries are medical doctor, dentist, nurse, midwife, emergency medical technician, paramedic, laboratory worker, health technician, medical secretary, cleaning staff, security, driver, technical staff, and others.

Ethical permission to use the data was obtained from the General Directorate of Public Health (E-49654233-604.02-240936446). The research data were analyzed using IBM SPSS 22 (Statistical Package for the Social Sciences) program. As a result, categorical variables were presented with numbers and percentages in the descriptive findings section. Fisher's chi-square test was applied in the analyses for the comparison of categorical variables.

## 3 Results

From the beginning of 2019 to the end of 2023, 68,563 occupational injuries were reported from healthcare organizations in Turkey.

In Turkey, 65.2% of healthcare workers are women. 21.2% of all healthcare staff are nurses, 14.7% are doctors and 7.7% are midwives. Of the healthcare workers who had occupational injuries, 64.5% were female. Among these injuries, 30.4% involved nurses, 8.7% involved doctors, and 3.8% involved midwives. While other healthcare personnel and othe staff make up 19.4 and 35.0% of healthcare workers in Turkey, respectively, 25.1 and 30.6% of occupational injuries occurred among these groups. There is a significant difference between the occupational groups of healthcare workers and workers who had occupational injuries (*p* < 0.05; Table 1). The difference is due to nurses and other healthcare personnel.

**Table 1:** Occupational injury prevalence according to gender and occupational groups of healthcare workers in Turkey.

	**Injured healthcare workers (%)**	**Total healthcare workers (%)**
**Gender**		
Female	64.5	65.2
Male	35.5	34.8
*p* = 0.917
**Occupational group**		
Nurses	30.4	21.1
Physicians	8.7	14.7
Midwives	3.8	7.7
Dentists	1.3	1.6
Pharmacists	0.1	0.5
Other health personnel^*^	25.1	19.4
Other personnel^**^	30.6	35.0
***p*** **<** **0.001**

^*^Other health personnel: nurse assistant, emergency medical technician, health technician, paramedic, caregiver, occupational safety specialist, occupational therapist, psychologist, chemist, etc.

^**^Other personnel: cleaning staff, medical secretary, security, driver, technical staff, laundry staff, software developer, treasurer, engineer, imam, barber, etc.

The bold value represents statistically significant differences.

**Figure 1:**
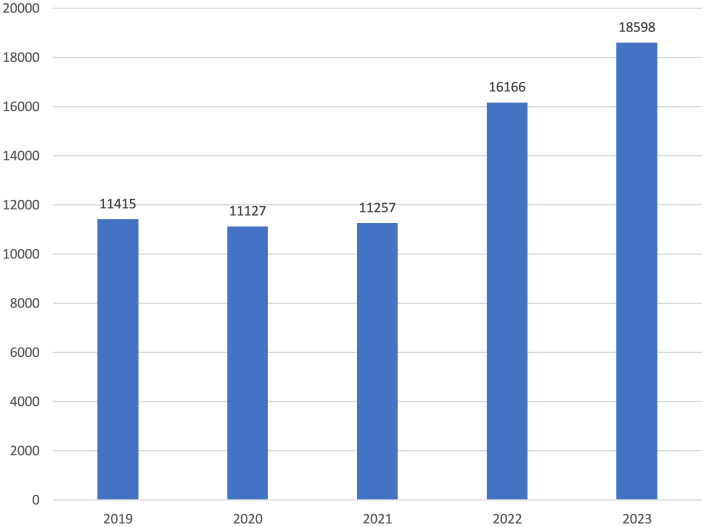
Occupational injuries by years (*n* = 68,563).

**Figure 2:**
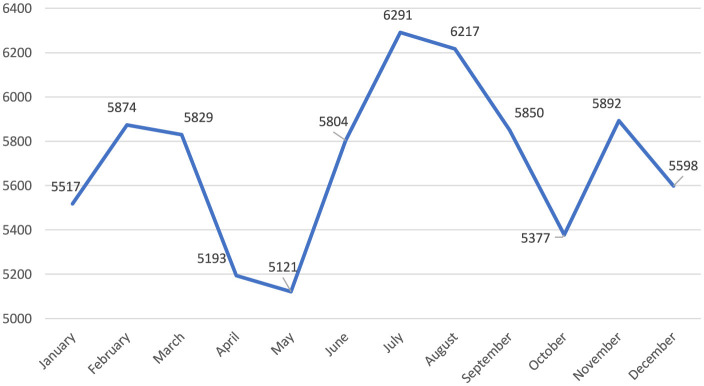
Monthly change in the number of occupational injuries.

**Figure 3:**
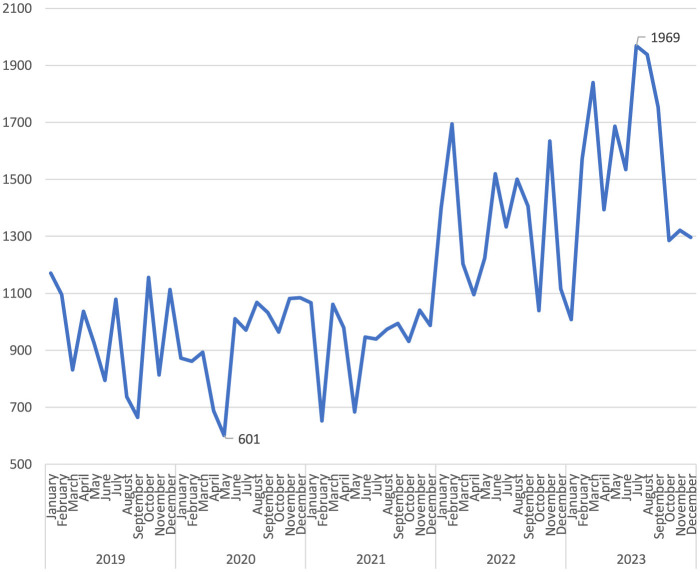
Changes in occupational injuries by years and months.

Notably, while there were similar numbers for the first 3 years, there was an increase of more than two-fifths in 2022 compared to the previous year. The upward trend continued in 2023 (Figure 1). When the distribution of the number of reported occupational injuries by month is examined, it is seen that the lowest numbers were in April and May, and the highest numbers were in July and August (Figure 2). In the 60 months of the study, the month with the lowest number of occupational injuries reported from healthcare institutions was May 2020 (*n* = 601), and the highest number was July 2023 (*n* = 1,969; Figure 3).

**Figure 4:**
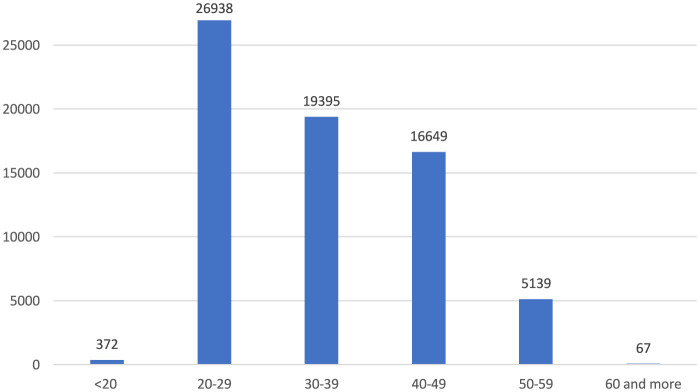
Age groups of occupational injuries among healthcare workers (*n* = 68,563).

**Figure 5:**
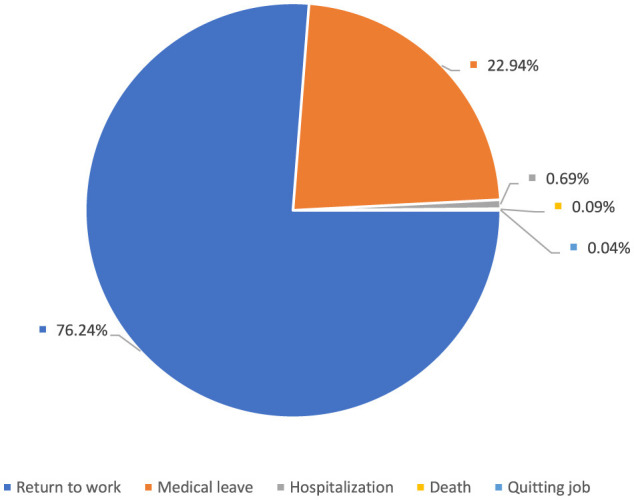
Consequences of occupational injuries among healthcare workers.

Among age groups, 20–29 account for 39.3%, 30–39 for 28.3%, and 40–49 for 24.3% (Figure 4). As a result of the reported occupational injuries, more than three-quarters of healthcare workers returned to work without loss of time and labor force, while nearly one-quarter had to undergo a medical report (Figure 5). In the 5 years, a total of 61 health workers lost their lives due to occupational injuries, and 24 had to quit their jobs.

**Figure 6:**
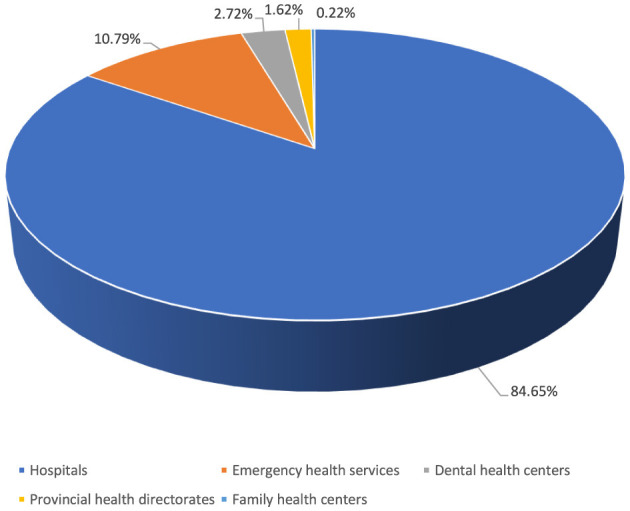
Health institutions reporting occupational injuries.

**Figure 7:**
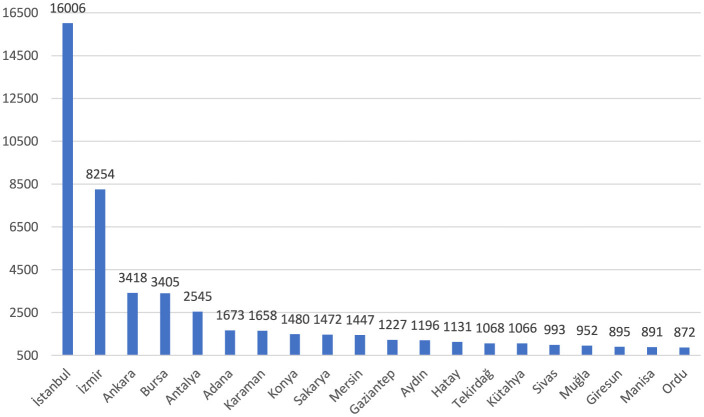
Cities with the most occupational injuries among healthcare workers (*n* = 68,563).

Of the reported occupational injuries, 84.7% were in hospitals and 10.8% were in first aid and emergency health services (Figure 6). Among the institutions with the highest number of occupational injuries were healthcare facilities in the non-metropolitan provinces of Karaman (7th), Kütahya (15th), Sivas (16th), and Giresun (18th). The highest number of occupational injuries was reported from health institutions in Istanbul, at 23.6% (Figure 7).

**Figure 8:**
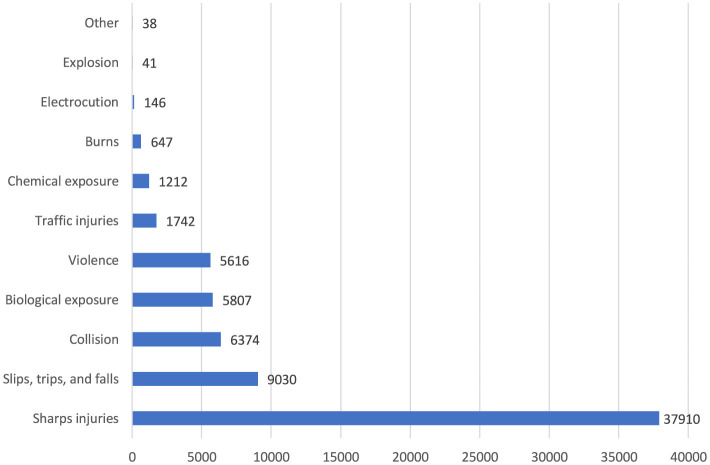
Types of occupational injuries among healthcare workers (*n* = 68,563). Other: animal attack, suicide, and heart attack.

**Figure 9:**
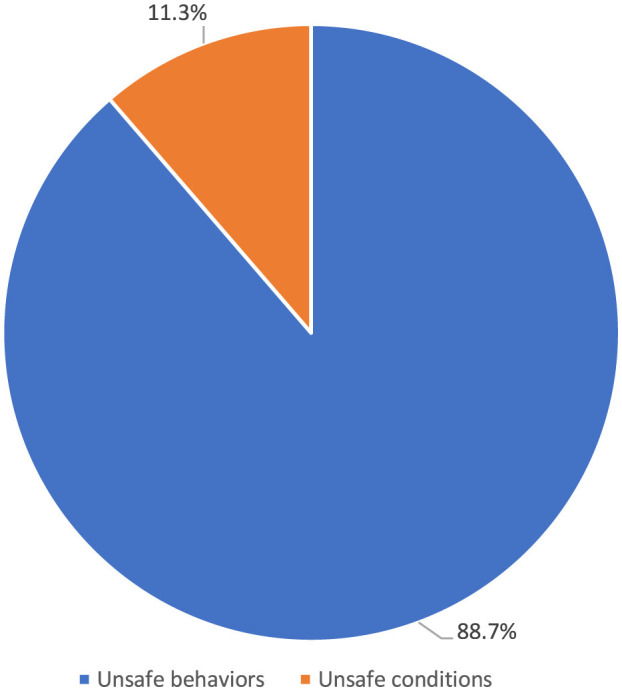
Probable causes of occupational injuries among health workers.

**Figure 10:**
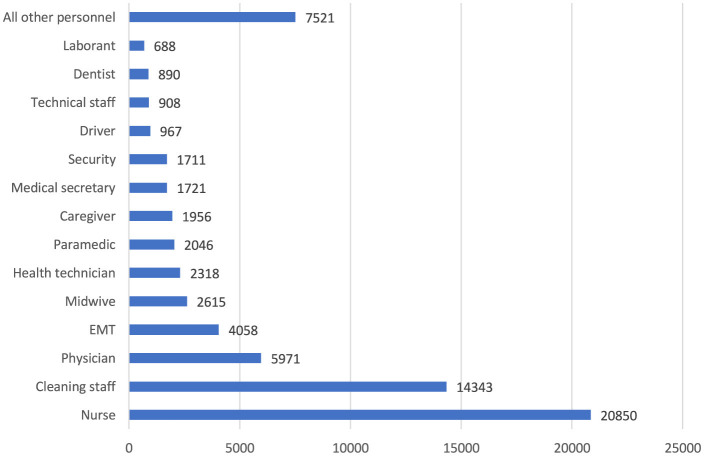
Job titles of healthcare workers with occupational injuries (*n* = 68,563). All other personnel: nurse assistant, pharmacist, laundry staff, software developer, occupational safety specialist, treasurer, engineer, occupational therapist, psychologist, chemist, imam, barber, etc.

The top five occupational injuries suffered by healthcare workers were sharps injuries (55.3%), slips, trips and falls (13.2%), bumps (9.3%), biological agent exposure (8.5%), and violence (8.2%; Figure 8). When the probable causes of occupational injuries are evaluated, unsafe behaviors and conditions have a frequency of 84.3 and 12.5%, respectively (Figure 9). Among occupational injury victims, 34.2% were nurses or midwives, 20.9% were cleaning staff, 8.9% were emergency medical technicians or paramedics, 8.7% were physicians, and 3.3% were health technicians (Figure 10).

## 4 Discussion

This study comprehensively analyzes occupational injuries among healthcare workers in Turkey over the last 5 years, reflecting the current state of occupational health and safety in the health sector. The findings indicate that healthcare workers are more frequently exposed to occupational injuries such as sharps injuries and slips, trips and falls. The higher incidence of occupational injuries among women is likely due to the fact that the majority of healthcare workers are women, and the higher prevalence of injuries among younger is also significant when considering occupational health and safety strategies. In Turkey, healthcare workers face occupational risks primarily due to structural challenges, including high workloads and insufficient emphasis on occupational health and safety practices in some institutions. This highlights the need for targeted strategies to enhance the occupational health and safety conditions for healthcare workers.

In Turkey, the number of occupational injuries reported by healthcare workers during the pandemic period, including 2020 and 2021, remained similar to 2019 levels, while a rising trend was observed in 2022 and 2023. During the pandemic, healthcare workers were likely under significant pressure due to increased workload and COVID-19-related emergencies, which may have impacted their ability to report occupational injuries promptly. In the United States, 440,044 healthcare workers contracted COVID-19, and among them, 1,469 deaths were reported, highlighting the severe toll of the pandemic on this population (13). This heavy impact, along with subsequent workforce shortages, may have influenced reporting behaviors, as some healthcare workers may not have returned to their jobs after recovery. Additionally, the pandemic brought increased attention to the challenging working conditions in healthcare, potentially leading to greater awareness about occupational safety and a rise in reported occupational injuries in 2022 and 2023. However, the effects of workforce shortages and increased health risks should be considered when interpreting these trends.

According to the results of our study, more than three-fourths of the healthcare workers returned to work immediately after the reported occupational injuries. In contrast, more than one-fifth did not continue to work for a certain period after receiving a medical report. Studies conducted in hospitals in Brazil and Portugal dealing with the outcomes of occupational injuries have very similar figures to our findings (14, 15). In a study conducted in a hospital in Turkey, one-tenth of the healthcare workers who had an occupational injury could not return to work for at least 1 day after the injury (16). In a study conducted in the United States of America, 49.4% of units providing direct patient care did not have any absence days (17). The extent to which employees can return to work immediately after occupational injuries varies depending on the type and severity of the injury and the general health status of the employee. The healthcare system and working conditions in different countries affect the rates of return to work after occupational injuries. Low absenteeism rates may be due to better support and recovery opportunities offered to healthcare workers. It may also be because the healthcare workers in the scope of the mentioned studies work in different centers. Thus, the risks they are exposed to and the occupational injuries they have experienced vary.

Nurses constitute approximately two-tenths of the healthcare workforce in Turkey (18), yet they represent more than three-tenths of healthcare workers who experience occupational injuries. This trend is consistent with international studies: Across various countries, nurses appear to be the group most vulnerable to occupational injuries (19–22). Several factors may explain this high rate of injuries among nurses. Nurses' frequent use of sharp instruments, such as needles and scalpels, adds to this risk, especially when factors like fatigue, fast-paced work, or lack of attention come into play. High-stress environments, such as emergency and intensive care units, often require sudden movements, increasing injury risks. Physical strain from patient care, especially when working with patients who have cognitive or behavioral issues, poses a significant risk. Additionally, nurses' intense workloads and long shifts can lead to mental and physical fatigue, further heightening the likelihood of occupational injuries in this group.

In our study, most reported occupational injuries (84.7%) occurred in hospitals, with emergency health services (10.8%) ranking second. Hospitals are considered the central point of healthcare services, and most healthcare personnel work in hospital environments. Occupational injuries occur more frequently in hospitals because healthcare workers are predominantly employed in hospitals, and the risks here are pervasive. In Turkey, the majority of healthcare services are provided in hospitals, where healthcare workers face more pronounced risks. Adopting higher standards for ensuring employee safety in hospitals is essential for improving occupational health and safety for healthcare workers in Turkey.

In this study, the top five occupational injuries suffered by healthcare workers were sharps injuries (55.3%), slips, trips, and falls (13.2%), bumps and collisions (9.3%), biological agent exposure (8.5%), and violence (8.2%). A research from a university hospital in Turkey indicated that 56.5% of employees who experienced occupational injuries had sharps injuries, 43.5% reported exposure to violence, and 28.3% encountered blood and body fluids (23). In an Indian study, needlestick injuries were the most common type of occupational injury, with a rate of 86% among healthcare workers (24). Similarly, a Polish study found that 36.9% of healthcare workers reported experiencing at least one needlestick injury during their career (25). In Finland, where safer sharps systems are more widely used, only 25.3% of healthcare workers reported needlestick injuries, and violence rates were lower at 5.4% (26). Although safer sharps systems are typically more expensive, they effectively reduce injury rates, suggesting that their broader adoption could mitigate sharps-related hazards. Differences in cultural factors and legal standards regarding occupational health and safety also influence the prevalence and reporting of occupational injuries, with some regions showing higher violence rates in healthcare settings.

Our study found that occupational injuries reported by health institutions decreased in April and May, which coincided with Ramadan in the study years. Changes in working hours, lunch breaks, and rest periods during Ramadan likely contributed to this decrease. May 2020, which saw the lowest number of injury reports across the years, also coincided with a curfew period in Turkey due to the COVID-19 pandemic. Therefore, the decrease in patient admissions during the pandemic, due to curfews and lockdowns, may have contributed to fewer reported occupational injuries, as healthcare workers were exposed to fewer high-risk situations.

Regarding health workforce density, provinces in Turkey can be divided into three categories, with 20 provinces in the first two categories. Accordingly, Istanbul and Ankara are in the first category. The second category includes Adana, Antalya, Aydın, Balıkesir, Bursa, Denizli, Diyarbakır, Gaziantep, Hatay, İzmir, Kayseri, Kocaeli, Konya, Manisa, Mersin, Nevşehir, Samsun, and Şanlıurfa. The remaining 61 provinces are in the third category (27). When occupational injuries of healthcare workers are analyzed, it is observed that although there are fewer healthcare workers, healthcare settings in İzmir have more notifications than those in Ankara. Despite being in the third category, it is noteworthy that notifications from Karaman, Sakarya, Tekirdağ, Kütahya, Sivas, Muğla, Giresun, and Ordu provinces are high.

On the other hand, Balıkesir, Denizli, Diyarbakır, Kayseri, Kayseri, Kocaeli, Nevşehir, Samsun, and Şanlıurfa are not among the top 20 cities where occupational injuries involving healthcare workers are reported the most, despite being in the top 20 in terms of health workforce density. Inadequate implementation of workplace safety practices in some healthcare institutions presents a significant barrier to protecting healthcare workers from occupational injuries. Strengthening safety standards, particularly in healthcare facilities outside major cities, is a critical step toward reducing injury rates.

Different dynamics can influence the reporting of occupational injuries. Firstly, there may be a difference in awareness of reporting occupational injuries. Some provinces might show greater sensitivity to occupational injuries, leading to more frequent reporting, while others could lack adequate reporting processes. There may also be differences in health services' structuring, management, and supervision. For instance, some provinces may have more efficient management of health facilities and a more sensitive approach to reporting occupational injuries, while others may be weaker in this respect. Recognizing imbalances in reporting or distributing occupational injuries can help better understand their causes and consequences. Conducting comprehensive research to identify and make visible the unreported occupational injuries would be appropriate.

In a study conducted in Turkey, 68% of healthcare workers with occupational injuries were female (28). Similarly, a Canadian study found that female workers had a significantly higher risk for all occupational injuries compared to their male counterparts [RR: 1.58 (1.24–2.01)] (29), and in Sweden, 67% of occupational injuries occurred in female healthcare workers (30). Our study also found that two-thirds of occupational injuries and illnesses occurred in women, aligning with the fact that 65% of healthcare workers in Turkey are female. Supporting this finding, research by another Turkish study also shows that female healthcare workers in Turkey face a significantly higher incidence of occupational injuries and illnesses compared to their male counterparts (31). This pattern reflects the concentration of women in high-risk roles like nursing and patient care, which involve physical demands and direct patient contact. Furthermore, healthcare workers typically work long and irregular hours, and women often face additional fatigue as they balance work and family responsibilities, further increasing the risk of injury. Additionally, the physiological and anatomical differences in women may make it more challenging to perform tasks such as heavy lifting and strenuous movements, contributing to a higher risk of occupational injuries.

According to our results, when the occupational injuries experienced by healthcare workers were analyzed in terms of age groups, it was observed that the highest prevalence was in the 20–29 age group. The prevalence gradually decreased with increasing age. In a systematic review by Salminen, most studies showed that young workers were exposed to occupational injuries more frequently than older workers (32). A study examining health service utilization due to work-related injuries among Canadian workers found that work-related injuries decreased with age (33). Young workers are generally not sufficiently informed about potential workplace hazards and lack experience by protecting themselves from risks. They can work faster and more dynamically in the work environment and this speed can sometimes lead to carelessness and distraction. In addition, young workers may be more willing to undertake hazardous tasks, which may increase the risk of occupational injuries. There are studies to support these arguments. In a retrospective study conducted in Turkey in which occupational injuries in healthcare workers were analyzed retrospectively, 41.0% of the injury victims had < 1 year of occupational experience, 37.7% had 1–5 years of occupational experience, and 10.0% had 6–10 years of occupational experience (34). In a Swedish study, shorter working experience and younger age were associated with unsafe attitudes of healthcare workers (35).

This study has some limitations. First, it is assumed that the occupational injury notifications are completely accurate and complete. However, it is possible that there are omissions or errors due to individual factors. On the other hand, the study benefited from a comprehensive data collection process conducted on a national scale over an extended period. Data from private health institutions were not included in the study, but the inclusion of data from all health institutions affiliated with the Ministry of Health across Turkey enhances the study's representativeness. Additionally, due to the retrospective nature of the study, it was not possible to investigate cause-and-effect relationships in depth, and the analysis was limited to observed associations. However, analyzing the data by age, gender, and job title provided insights into the specific risks and needs of different demographic groups.

## 5 Conclusion

Our study, which examined healthcare workers' exposure to occupational injuries in Turkey, highlights the prevalence and variety of occupational risks in the healthcare sector. Our findings show that female healthcare workers experience occupational injuries at a higher rate than their male counterparts. Given that nurses and midwives are the most affected groups, with nurses being the profession most frequently involved in occupational injuries, implementing targeted safety measures for these professions is crucial to improve occupational safety.

Unsafe behaviors significantly contribute to injuries, addressing underlying environmental and organizational factors is important. This involves ensuring safe working environments in healthcare facilities through structural and procedural measures to reduce occupational risks. For the wellbeing of healthcare workers and the quality of healthcare services, establishing comprehensive safety protocols and continually improving workplace conditions are essential. Strategic plans should focus on creating safer working conditions, preventing occupational injuries, and minimizing their impact. These measures are vital to maintaining and enhancing the effectiveness of healthcare services.

## Data Availability

The datasets presented in this article are not readily available because the Ministry of Health prohibits the sharing of its national database containing health data with third parties. Requests to access the datasets should be directed to İrem Medeni—irem.medeni@saglik.gov.tr.
